# Supplementary material for: Phylodynamics of H5N1 Highly Pathogenic Avian Influenza in Europe, 2005–2010: Potential for Molecular Surveillance of New Outbreaks
Source: Viruses. 2015 Jun 23;7(6):3310–28. doi: 10.3390/v7062773 (PMC4488740; doi:10.3390/v7062773)
Supplement: Supplementary File 1 [file viruses-07-02773-s001.pdf]

Supplementary Information

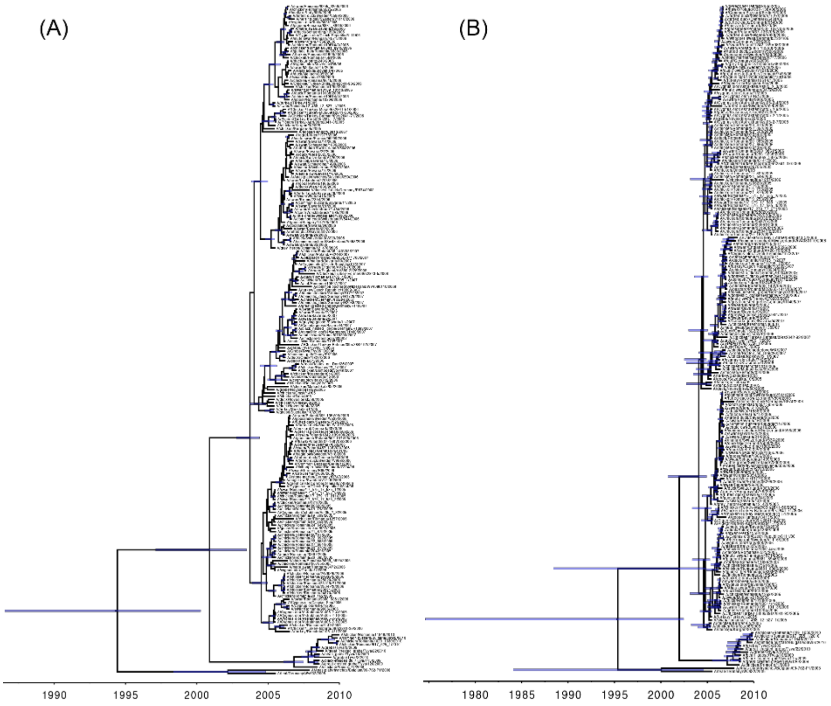

Figure S1. Maximum clade credibility (MCC) trees for H5N1 HPAIV hemagglutinin (HA) and neauraminidase (NA) gene regions (**A**,**B**) respectively, Branch lengths are rendered proportional to absolute time (see timescales). Nodes correspond to median ages and the blue horizontal bars at nodes represent the corresponding 95% HPDs for divergence-time estimates.

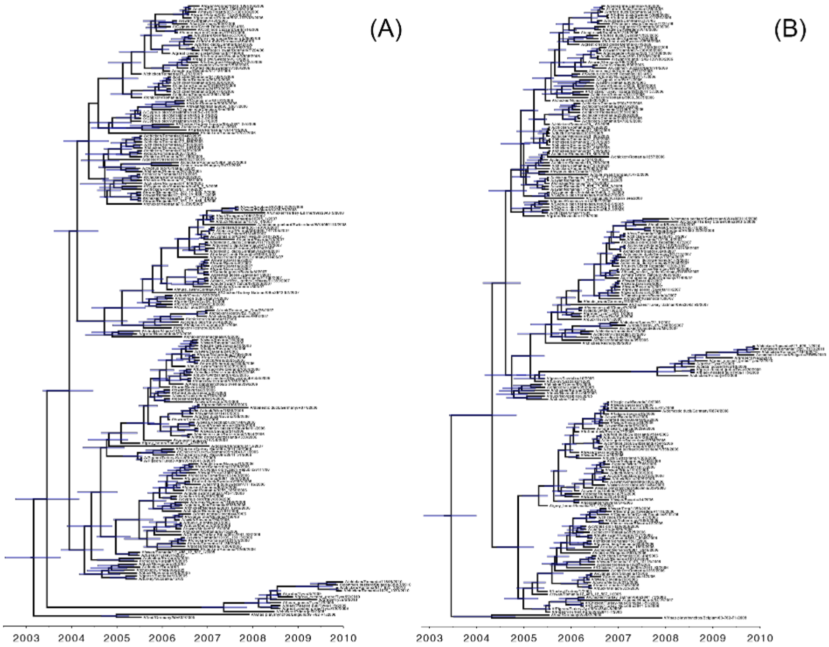

Figure S2. Maximum clade credibility (MCC) trees for H5N1 HPAIV hemagglutinin (HA) and neauraminidase (NA) gene regions (**A**,**B**) respectively, Branch lengths are rendered proportional to absolute time (see timescales). Nodes correspond to median ages and the blue horizontal bars at nodes represent the corresponding 95% HPDs for divergence-time estimates.

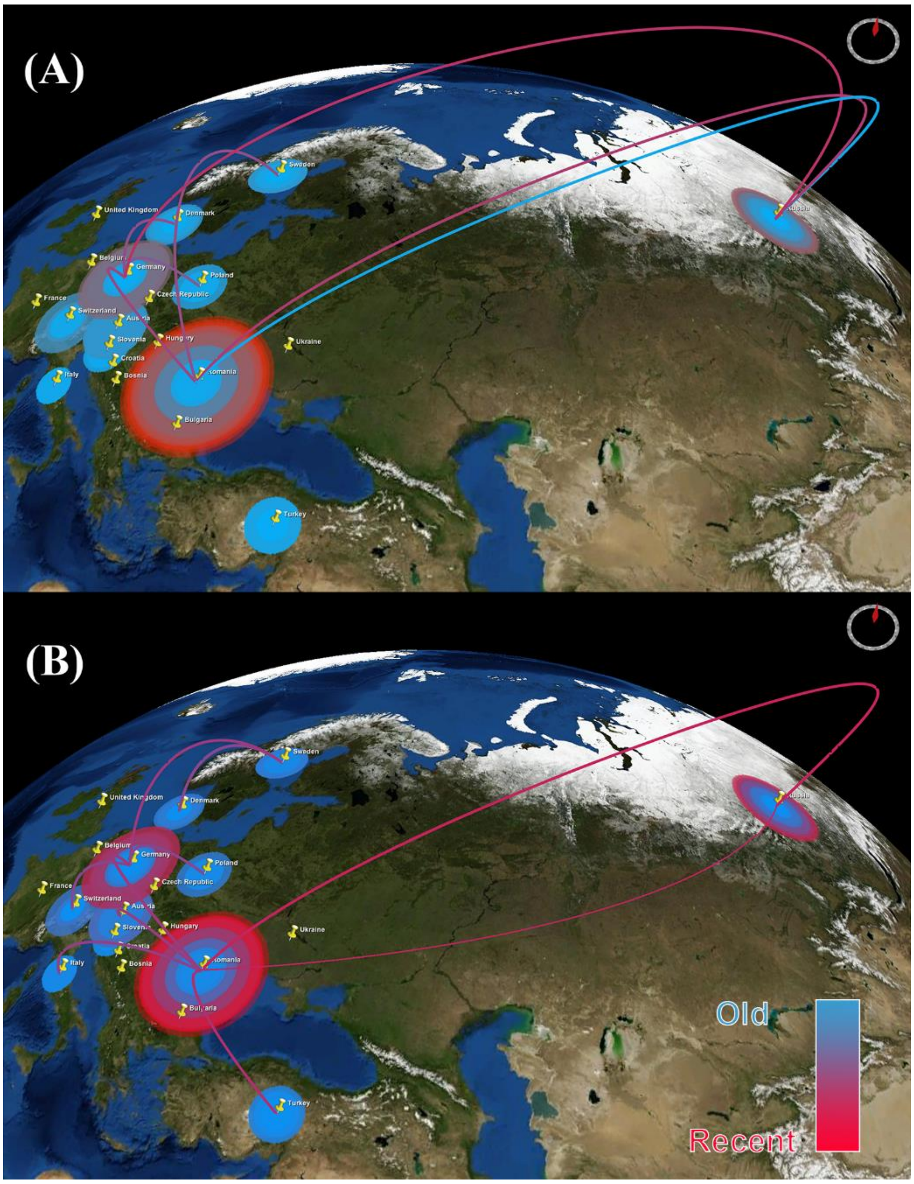

Figure S3. Temporal dynamics of the spatial diffusion of H5N1 HPAIV**.** The figure consists of snap shots of the dispersal pattern of avian influenza between May 2005 and June 2010. Lines between locations represent branches in the MCC tree along which spatial transmission occurs. The diameters of circles are proportional to square root of the number of MCC branches maintaining a particular location state at each time-point. The blue and red color gradients reflect the relative age of the transitions for each gene (older-recent, respectively). A: HA gene. B: NA gene. The maps are based on satellite pictures available in NASA World Wind (http://worldwind.arc.nasa.gov/java/).

File S1. Temporal dynamics of the spatial diffusion of H5N1 HPAIV based on the HA gene.

The KML file demonstrates the dispersal pattern of avian influenza between May 2005 and June 2010. Lines between locations represent branches in the MCC tree along which spatial transmission occurs. The diameters of circles are proportional to square root of the number of MCC branches maintaining a particular location state at each time-point. The blue and red color gradients reflect the relative age of the transitions for HA gene (older-recent, respectively).

Table S1. GISAID database Accession numbers and isolate names matched with other public databases used in this study for both HA and NA gene sequences isolated from Europe and Russia, with the exception of sequences isolated from Romania, which has been published elsewhere [1].

EpiFlu ID	Isolate Name
EPI_ISL_66178	A/coot/Voecklabruck/1585/2006
EPI_ISL_66194	A/duck/Leibnitz/243/2006
EPI_ISL_66195	A/duck/Mellach/335/2006
EPI_ISL_66196	A/duck/Schaerding/1398/2006
EPI_ISL_66199	A/duck/Wels/2025/2006
EPI_ISL_66200	A/duck/Wien/1836/2006
EPI_ISL_66201	A/egret/Wien/1977/2006
EPI_ISL_66202	A/goose/Wien/1966/2006
EPI_ISL_66219	A/swan/Krems/2354/2006
EPI_ISL_66220	A/swan/Krems/2547/2006
EPI_ISL_66221	A/swan/Krems/2675/2006
EPI_ISL_66222	A/swan/Laakirchen/2703/2006
EPI_ISL_66223	A/swan/Mellach/215/2006
EPI_ISL_66225	A/swan/Perg/1358/2006
EPI_ISL_66226	A/swan/Schaerding/1499/2006
EPI_ISL_66227	A/swan/Schaerding/1806/2006
EPI_ISL_66228	A/swan/Schwechat/2538/2006
EPI_ISL_66229	A/swan/Voecklabruck/1484/2006
EPI_ISL_66230	A/swan/Wien/1410/2006
EPI_ISL_66231	A/swan/Wien/2323/2006
EPI_ISL_74836	A/Anas platyrhynchos/Belgium/09-762-P1/2008
EPI_ISL_64083	A/Cygnus olor/BIH/1/2006
EPI_ISL_105998	A/common buzzard/Bulgaria/38WB/2010
EPI_ISL_6386	A/cygnus olor/Croatia/1/2005
EPI_ISL_15685	A/Cygnus olor/Czech Republic/6111/06
EPI_ISL_15689	A/Cygnus olor/Czech Republic/10814/06
EPI_ISL_15690	A/turkey/Czech Republic/10309-3/07
EPI_ISL_15691	A/chicken/Czech Republic/11242-38/07
EPI_ISL_15692	A/Cygnus olor/Czech Republic/10732/07
EPI_ISL_15693	A/Cygnus olor/Czech Republic/10662/06
EPI_ISL_69720	A/buzzard/Denmark/6370/06
EPI_ISL_69730	A/tufted duck/Denmark/6431/06
EPI_ISL_69724	A/tufted duck/Denmark/6540/06
EPI_ISL_69725	A/peregrine/Denmark/6632/06
EPI_ISL_69731	A/grey lag goose/Denmark/6692/06
EPI_ISL_69732	A/whooper swan/Denmark/7224/06
EPI_ISL_69722	A/whooper swan/Denmark/7275/06
EPI_ISL_69721	A/great crested grebe/Denmark/7498/06
EPI_ISL_2988	A/mute swan/France/06299/2006
EPI_ISL_31666	A/mute swan/France/070203/2007
EPI_ISL_74112	A/teal/Germany/Wv632/2005

Table S1. *Cont.*

EpiFlu ID	Isolate Name
EPI_ISL_10142	A/swan/Germany/R65/2006
EPI_ISL_2985	A/stone marten/Germany/R747/2006
EPI_ISL_10142	A/swan/Germany/R65/2006
EPI_ISL_10333	A/cat/Germany/606/2006
EPI_ISL_68227	A/swan/Bavaria/25/2006
EPI_ISL_68226	A/swan/Bavaria/24/2006
EPI_ISL_68225	A/swan/Bavaria/23/2006
EPI_ISL_68228	A/tufted duck/Bavaria/26/2006
EPI_ISL_11353	A/falcon/Bavaria/15/2006
EPI_ISL_11359	A/swan/Bavaria/21/2006
EPI_ISL_11355	A/swan/Bavaria/17/2006
EPI_ISL_11354	A/swan/Bavaria/16/2006
EPI_ISL_11352	A/swan/Bavaria/14/2006
EPI_ISL_11350	A/mute swan/Bavaria/12/2006
EPI_ISL_11360	A/great crested grebe/Bavaria/22/2006
EPI_ISL_11358	A/goosander/Bavaria/20/2006
EPI_ISL_11356	A/goosander/Bavaria/18/2006
EPI_ISL_11351	A/buzzard/Bavaria/13/2006
EPI_ISL_11348	A/eagle owl/Bavaria/10/2006
EPI_ISL_11357	A/goldeneye duck/Bavaria/19/2006
EPI_ISL_11349	A/common buzzard/Bavaria/11/2006
EPI_ISL_68235	A/swan/Bavaria/33/2006
EPI_ISL_68234	A/swan/Bavaria/32/2006
EPI_ISL_68231	A/swan/Bavaria/29/2006
EPI_ISL_68230	A/swan/Bavaria/28/2006
EPI_ISL_68233	A/great crested grebe/Bavaria/31/2006
EPI_ISL_68236	A/goosander/Bavaria/34/2006
EPI_ISL_68232	A/common pochard/Bavaria/30/2006
EPI_ISL_68229	A/tufted duck/Bavaria/27/2006
EPI_ISL_3011	A/mute_swan/Germany/R1359/07
EPI_ISL_24700	A/mallard/Bavaria/10/2007
EPI_ISL_27795	A/domestic_goose/Germany/R1400/2007
EPI_ISL_27769	A/domestic_duck/Germany/R2048/2007
EPI_ISL_27768	A/domestic_duck/Germany/R1779/2007
EPI_ISL_27797	A/great crested grebe/Germany/R1406/07
EPI_ISL_68254	A/Canada goose/Bavaria/4/2007
EPI_ISL_68255	A/swan/Bavaria/5/2007
EPI_ISL_68252	A/swan/Bavaria/2/2007
EPI_ISL_68253	A/mute swan/Bavaria/3/2007
EPI_ISL_63430	A/black_nested_ grebe/Germany/R1393/2007
EPI_ISL_68260	A/greylag goose /Bavaria/11/2007
EPI_ISL_68259	A/swan/Bavaria/9/2007
EPI_ISL_68258	A/swan/Bavaria/8/2007
EPI_ISL_68257	A/swan/Bavaria/7/2007
EPI_ISL_68256	A/swan/Bavaria/6/2007

Table S1. *Cont.*

EpiFlu ID	Isolate Name
EPI_ISL_68261	A/wild duck/Bavaria/12/2007
EPI_ISL_27767	A/domestic duck/Germany/R1772/2007
EPI_ISL_27773	A/chicken/Germany/R3234/2007
EPI_ISL_27771	A/chicken/Germany/R3272/2007
EPI_ISL_27772	A/chicken/Germany/R3294/2007
EPI_ISL_2963	A/whooper swan/Germany/R88/06
EPI_ISL_10140	A/mallard/Bavaria/1/2006
EPI_ISL_25455	A/domestic duck/Germany/R874/2008
EPI_ISL_32413	A/duck/Germany/R854/2008
EPI_ISL_25455	A/domestic duck/Germany/R874/2008
EPI_ISL_31503	A/mute swan/Hungary/3472/2006
EPI_ISL_31502	A/mute swan/Hungary/4571/2006
EPI_ISL_31501	A/goose/Hungary/14756/2006
EPI_ISL_11425	A/goose/Hungary/3413/2007
EPI_ISL_63515	A/goose/Hungary/2823/2/2007
EPI_ISL_6383	A/mallard/Italy/835/2006
EPI_ISL_21034	A/cygnus olor/Italy/742/2006
EPI_ISL_6891	A/cygnus olor/Italy/808/2006
EPI_ISL_27762	A/chicken/Poland/R3248/2007
EPI_ISL_27763	A/turkey/Poland/R3249/2007
EPI_ISL_20922	A/swan/Poland/1242-139V08/2006
EPI_ISL_20921	A/hawk/Poland/937-138V08/2006
EPI_ISL_20920	A/swan/Poland/467-136V08/2006
EPI_ISL_20919	A/goosander/Poland/502-137V08/2006
EPI_ISL_20918	A/buzzard/Poland/MB266B-141V08/2007
EPI_ISL_20917	A/chicken/Poland/38-140V08/2007
EPI_ISL_20916	A/swan/Poland/305-135V08/2006
EPI_ISL_11388	A/goose/Krasnoozerskoe/627/2005
EPI_ISL_11387	A/goose/Suzdalka/10/2005
EPI_ISL_10106	A/Cygnus olor/Astrakhan/Ast05-2-9/2005
EPI_ISL_10103	A/Cygnus olor/Astrakhan/Ast05-2-1/2005
EPI_ISL_10101	A/Cygnus olor/Astrakhan/Ast05-2-8/2005
EPI_ISL_10051	A/Cygnus olor/Astrakhan/Ast05-2-7/2005
EPI_ISL_10050	A/Cygnus olor/Astrakhan/Ast05-2-4/2005
EPI_ISL_11389	A/turkey/Suzdalka/12/05
EPI_ISL_9768	A/grebe/Novosibirsk/29/2005
EPI_ISL_11391	A/chicken/Tula/4/05
EPI_ISL_11386	A/chicken/Omsk/14/05
EPI_ISL_11385	A/chicken/Suzdalka/06/05
EPI_ISL_10137	A/chicken/Kurgan/05/2005
EPI_ISL_10138	A/duck/Kurgan/08/2005
EPI_ISL_9769	A/duck/Novosibirsk/56/2005
EPI_ISL_10121	A/Cygnus olor/Astrakhan/Ast05_2_10/2005
EPI_ISL_10053	A/Cygnus olor/Astrakhan/Ast05_2_5/2005
EPI_ISL_10052	A/Cygnus olor/Astrakhan/Ast05_2_6/2005

Table S1. *Cont.*

EpiFlu ID	Isolate Name
EPI_ISL_10008	A/Cygnus olor/Astrakhan/Ast05_2_2/2005
EPI_ISL_10048	A/Cygnus olor/Astrakhan/Ast05_2_3/2005
EPI_ISL_75725	A/Cygnus olor/Caspian Sea/2006
EPI_ISL_11390	A/chicken/Krasnodar/123/06
EPI_ISL_64332	A/chicken/Reshoty/02/2006
EPI_ISL_10543	A/grebe/Tyva/Tyv06_1/2006
EPI_ISL_10440	A/Grebe/Tyva/Tyv06_8/2006
EPI_ISL_10435	A/grebe/Tyva/Tyv06_2/06
EPI_ISL_64356	A/duck/Omsk/1822/2006
EPI_ISL_14423	A/chicken/Rostov/22_1/2007
EPI_ISL_63440	A/chicken/Domodedovo/MK/2007
EPI_ISL_11823	A/chicken/Moscow/2/2007
EPI_ISL_13577	A/chicken/Krasnodar/300/07
EPI_ISL_13848	A/Cygnus_cygnus/Krasnodar/329/07
EPI_ISL_20040	A/rook/Rostov_on_Don/26/2007
EPI_ISL_15359	A/pigeon/Rostov_on_Don/6/2007
EPI_ISL_14419	A/chicken/Rostov_on_Don/35/2007
EPI_ISL_15357	A/starling/Rostov_on_Don/39/2007
EPI_ISL_15358	A/muscovy duck/Rostov_on_Don/51/2007
EPI_ISL_63010	A/chicken/Primorje/1/2008
EPI_ISL_32668	A/bean_goose/Tyva/10/2009
EPI_ISL_32667	A/grebe/Tyva/3/2009
EPI_ISL_80265	A/great_crested_grebe/Tyva/22/2010
EPI_ISL_84604	A/grebe/Tyva/2/2010
EPI_ISL_31936	A/black-headed gull/Tyva/115/2009
EPI_ISL_28437	A/chicken/Primorsky/85/2008
EPI_ISL_9967	A/chicken/Kurgan/3/2005
EPI_ISL_64866	A/duck/Novosibirsk/02/05
EPI_ISL_10436	A/duck/Tuva/01/2006
EPI_ISL_10361	A/chicken/Krasnodar/01/2006
EPI_ISL_10360	A/chicken/Mahachkala/05/2006
EPI_ISL_64343	A/common gull/Chany/P/2006
EPI_ISL_31937	A/great crested grebe/Tyva/120/2009
EPI_ISL_15688	A/Mergus albellus/Slovakia/Vh212/2006
EPI_ISL_27786	A/Peregrine falcon/Slovakia/Vh242/2006
EPI_ISL_6414	A/swan/Slovenia/760/2006
EPI_ISL_27791	A/cygnus olor/Slovenia/156/06
EPI_ISL_27792	A/Ardea cinerea/Slovenia/185/06
EPI_ISL_27793	A/Anas platyrhynchos/Slovenia/359/2006
EPI_ISL_27794	A/Anas acuta/Slovenia/470/06
EPI_ISL_13230	A/tufted duck/Sweden/V526/2006
EPI_ISL_13231	A/goosander/Sweden/V539/2006
EPI_ISL_13233	A/tufted duck/Sweden/V599/2006
EPI_ISL_24705	A/eagle owl/Sweden/V618/2006
EPI_ISL_13240	A/smew/Sweden/V820/2006

Table S1. *Cont.*

EpiFlu ID	Isolate Name
EPI_ISL_24704	A/eagle owl/Sweden/V1218/2006
EPI_ISL_13249	A/herring gull/Sweden/V1116/2006
EPI_ISL_13247	A/tufted duck/Sweden/V1027/2006
EPI_ISL_13246	A/tufted duck/Sweden/V998/2006
EPI_ISL_13245	A/canada goose/Sweden/V978/2006
EPI_ISL_13241	A/mute swan/Sweden/V827/2006
EPI_ISL_69212	A/common pochard/Switzerland/WV4080110/2008
EPI_ISL_11231	A/common coot/Switzerland/V544/2006
EPI_ISL_11917	A/tufted duck/Switzerland/V504/2006
EPI_ISL_12822	A/mute swan/Switzerland/V68/2006
EPI_ISL_12823	A/little grebe/Switzerland/V330/2006
EPI_ISL_12824	A/duck/Switzerland/V389/2006
EPI_ISL_12825	A/duck/Switzerland/V426/2006
EPI_ISL_12826	A/duck/Switzerland/V487/2006
EPI_ISL_12827	A/common pochard/Switzerland/V505/2006
EPI_ISL_12828	A/mallard/Switzerland/V537/2006
EPI_ISL_12829	A/common pochard/Switzerland/V558/2006
EPI_ISL_12830	A/common pochard/Switzerland/V592/2006
EPI_ISL_12831	A/common pochard/Switzerland/V762/2006
EPI_ISL_10340	A/chicken/Crimea/08/2005
EPI_ISL_103335	A/swan/England/AV26-70/2008
EPI_ISL_103350	A/swan/England/AV380-2326/2008
EPI_ISL_99850	A/Common Buzzard/Berlin/1/2006
[1]	A/chicken/Romania/1210/2005
[1]	A/grey_heron/Romania/12_478/2005
[1]	A/swan/Romania/12_488_12_527_1/2005
[1]	A/chicken/Romania/17_279_17_311/2005
[1]	A/swan/Romania/17_575_17_585_2/2005
[1]	A/swan/Romania/17_575_17_585_3/2005
[1]	A/swan/Romania/17_575_17_585_1/2005
[1]	A/chicken/Romania/19_809/2005
[1]	A/chicken/Romania/20_162/2005
[1]	A/chicken/Romania/19_793/2005
[1]	A/chicken/Romania/19_253/2005
[1]	A/chicken/Romania/20_085/2005
[1]	A/chicken/Romania/20627/2005
[1]	A/chicken/Romania/20439/2005
[1]	A/duck/Romania/20367/2005
[1]	A/chicken/Romania/20620/2005
[1]	A/chicken/Romania/20261/2005
[1]	A/chicken/Romania/20447/2005
[1]	A/chicken/Romania/20640/2005
[1]	A/chicken/Romania/21_879/2005
[1]	A/chicken/Romania/23_870/2005
[1]	A/chicken/Romania/24_649/2005

Table S1. *Cont.*

EpiFlu ID	Isolate Name
[1]	A/chicken/Romania/26705/2005
[1]	A/chicken/Romania/22_668/2005
[1]	A/chicken/Romania/22_092/2005
[1]	A/turkey/Romania/27277_27281/2005
[1]	A/chicken/Romania/1269/2006
[1]	A/chicken/Romania/1257/2006
[1]	A/chicken/Romania/27_664/2006
[1]	A/chicken/Romania/5869_5875/2006
[1]	A/chicken/Romania/6141/2006
[1]	A/chicken/Romania/6525/2006
[1]	A/chicken/Romania/6615/2006
[1]	A/chicken/Romania/7561/2006
[1]	A/chicken/Romania/8881_8946/2006
[1]	A/pigeon/Romania/8881_8946/2006
[1]	A/guinea_hen/Romania/8338/2006
[1]	A/swan/Romania/10109_10113/2006
[1]	A/swan/Romania/9906/2006
[1]	A/swan/Romania/9255_9256/2006
[1]	A/swan/Romania/9860_9861/2006
[1]	A/chicken/Romania/10480/2006
[1]	A/chicken/Romania/10790/2006
[1]	A/goose/Romania/10204/2006
[1]	A/chicken/Romania/10474/2006
[1]	A/turkey/Romania/10614/1/2006
[1]	A/chicken/Romania/10614/2/2006
[1]	A/chicken/Romania/10614/3/2006
[1]	A/chicken/Romania/10614/4/2006
[1]	A/turkey/Romania/11896/2006
[1]	A/duck/Romania/10138/2006
[1]	A/chicken/Romania/24932/2/2006
[1]	A/chicken/Romania/24870/2006
[1]	A/chicken/Romania/24792/2006
[1]	A/chicken/Romania/25044/2/2006
[1]	A/chicken/Romania/25047/2/2006
[1]	A/chicken/Romania/24402/2006
[1]	A/chicken/Romania/24782/6/2006
[1]	A/chicken/Romania/24842/2006
[1]	A/chicken/Romania/24123/2006
[1]	A/chicken/Romania/24425/2006
[1]	A/chicken/Romania/24776/2006
[1]	A/chicken/Romania/24000/2006
[1]	A/chicken/Romania/25020/2006
[1]	A/chicken/Romania/23196/P1/2006
[1]	A/chicken/Romania/23991/2006
[1]	A/duck/Romania/10705_4/2007

Table S1. *Cont.*

EpiFlu ID	Isolate Name
[1]	A/duck/Romania/10705_5/2007
[1]	A/chicken/Romania/10705_1/2007
[1]	A/chicken/Romania/10705_2/2007
[1]	A/cat/Romania/10807/2007
[1]	A/chicken/Romania/611_616_1/2010
[1]	A/chicken/Romania/611_616_2/2010
[1]	A/chicken/Romania/11848/2010
[1]	A/chicken/Romania/617_625_1/2010
[1]	A/chicken/Romania/617_625_2/2010
[1]	A/chicken/Romania/941/2010
[1]	A/chicken/Romania/1029_1030/2010

Table S2. Summary profile of H5N1 HPAIV sequences isolated from reported outbreaks in Europe and the Russian Federation between May, 2005 and June, 2010 (n = 277).

Country	N.seq	Collection date	Host type
Austria	20	February 2006-April 2006	Wild
Belgium	1	November 2008	Wild
Bosnia	1	February 2006	Wild
Bulgaria	1	March 2010	Wild
Croatia	1	October 2005	Wild
Czech Republic	6	March 2006–July 2007	Wild & domestic
Denmark	8	March 2006–April 2006	Wild
France	2	February 2006-June 2007	Wild
Germany	57	November 2005–October 2008	Wild, domestic, & Other
Hungary	5	February 2006–January 2007	Wild
Italy	3	February 2006	Wild
Poland	9	March 2006–December 2007	Wild & domestic
Romania	78	October 2005–March 2010	Wild, domestic, & Other
Russia	51	July 2005–January 2010	Wild & domestic
Slovakia	2	February 2006	Wild
Slovenia	5	February 2006–March 2006	Wild
Sweden	11	March 2006–April 2006	Wild
Switzerland	13	February 2006–February 2008	Wild
Ukraine	1	May 2005	Domestic
UK	2	January 2008	Wild

Table S3. Candidate phylogeographic models for HA and NA gene segments explored for relative fit.

Model	Sites	Discrete-Traits	Branch Rates	Trees
1	GTR + GAMMA	Symmetric reversible	Strict clock	Coalescent: GMRF Bayesian Skyride
2			UCLN	
3			UCED	
4		Asymmetric irreversible	Strict clock	
5			UCLN	
6			UCED	

Table S4. Bayes factor test for non-zero rates for location (country) state model for both HA and NA genes.

Gene	BF	Between	
HA	77823.6	Russia	Romania
HA	77823.6	Germany	Austria
HA	77823.6	Germany	Czech Republic
HA	8639.4	Turkey	Romania
HA	317.0	Romania	Germany
HA	274.4	Poland	Germany
HA	90.6	Romania	Hungary
HA	87.4	Romania	Italy
HA	48.5	Ukraine	Russia
HA	46.4	Switzerland	Germany
HA	43.4	Germany	France
HA	16.0	Romania	Bosnia
HA	13.9	Sweden	Romania
HA	11.9	Romania	Bulgaria
HA	11.1	Romania	Croatia
HA	9.8	Slovenia	Austria
HA	7.7	Sweden	Denmark
HA	7.0	Germany	Denmark
NA	77823.6	Romania	Germany
NA	2984.9	Russia	Romania
NA	2502.1	Turkey	Romania
NA	441.2	Germany	Czech Republic
NA	409.8	Switzerland	Germany
NA	240.0	Sweden	Denmark
NA	190.9	Romania	Hungary
NA	149.2	Poland	Germany
NA	108.2	Germany	France
NA	87.0	Ukraine	Russia
NA	57.9	Germany	Austria
NA	27.5	Romania	Italy
NA	16.3	Romania	Bulgaria
NA	15.4	Slovenia	Austria
NA	15.3	Sweden	Germany

Table S4. *Cont.*

Gene	BF	Between	Gene
NA	11.1	Romania	Bosnia
NA	7.6	United Kingdom	Germany
NA	6.9	Romania	Croatia

Significant routes had a Bayes Factor (BF) of at least 6

1. Alkhamis, M.; Perez, A.; Batey, N.; Howard, W.; Watson, S.; Baillie, G.; Franz, S.; Focosi-Snyman, R.; Onita, I.; Cioranu, R.; *et al.* Modeling the association of space, time, and host species with variation of the HA, NA, and NS genes of H5N1 highly pathogenic avian influenza viruses isolated from birds in Romania in 2005–2007. *Avian Dis.* **2013**, *57*, 612–621.
